# Evaluation of the Clinical, Biochemical, Neurological, and Genetic Presentations of Glutaric Aciduria Type 1 in Patients From China

**DOI:** 10.3389/fgene.2021.702374

**Published:** 2021-07-07

**Authors:** Huishu E., Lili Liang, Huiwen Zhang, Wenjuan Qiu, Jun Ye, Feng Xu, Zhuwen Gong, Xuefan Gu, Lianshu Han

**Affiliations:** Department of Pediatric Endocrinology and Genetic, Shanghai Institute for Pediatric Research, Xinhua Hospital Affiliated to Shanghai Jiao Tong University School of Medicine, Shanghai, China

**Keywords:** glutaric aciduria type 1, glutaryl-CoA dehydrogenase, glutarylcarnitine, capryloylcarnitine, GCDH gene

## Abstract

**Purpose:**

To characterize the phenotypic and genotypic variations associated with Glutaric aciduria type 1 (GA1) in Chinese patients.

**Methods:**

We analyzed the clinical, neuroradiological, biochemical, and genetic information from 101 GA1 patients in mainland China.

**Results:**

20 patients were diagnosed by newborn screening and the remaining 81 cases were identified following clinical intervention. Macrocephaly was the most common presentation, followed by movement disorders and seizures. A total of 59 patients were evaluated by brain MRI and 58 patients presented with abnormalities, with widening of the sylvian fissures being the most common symptom. The concentration of glutarylcarnitine in the blood, glutarylcarnitine/capryloylcarnitine ratio, and urine levels of glutaric acid were increased in GA1 patients and were shown to decrease following intervention. A total of 88 patient samples were available for genotyping and 74 variants within the *GCDH* gene, including 23 novel variants, were identified. The most common variant was c.1244-2A > C (18.4%) and there were no significant differences in the biochemical or clinical phenotypes described for patients with the four most common variants: c.1244-2A > C, c.1064G > A, c.533G > A, and c.1147C > T. Patients identified by newborn screening had better outcomes than clinical patients.

**Conclusion:**

Our findings expand the spectrum of phenotypes and genotypes for GA1 in Chinese populations and suggest that an expanded newborn screening program using tandem mass spectrometry may facilitate the early diagnosis and treatment of this disease, improving clinical outcomes for patients in China.

## Introduction

Glutaric aciduria type 1 (GA1, OMIM #231670) is a rare autosomal recessive disorder caused by the variants in glutaryl-CoA dehydrogenase (GCDH) gene, leading to a marked decrease in glutaryl- GCDH activity, which will resulting in the accumulation of glutaric acid (GA), 3-hydroxyglutaric acid (3-OH-GA), and glutarylcarnitine (C5DC) in various tissues, especially the brain (Kolker et al., 2006). The *GCDH* gene is mapped to chromosome 19p13.2 and spans approximately 7 kb of genomic DNA. The worldwide incidence of GA1 is estimated to be 1/110,000 (Boy et al., 2017b); however, prevalence rates are higher in certain genetically homogeneous communities such as the Old Order Amish of Lancaster County, Pennsylvania (1/300–1/400), and the aboriginal Ojibway-Cree Indians of Northern Canada (1/300) where common variants are often found in homozygous states (Haworth et al., 1991; Morton et al., 1991). The nationwide prevalence of GA1 is between 1/171,411 and 1/52,078 in China, accounting for 7.6% of the organic acidemia (Han et al., 2015; Lin et al., 2019; Yang et al., 2020).

Glutaric aciduria type 1 is further divided into two biochemical subgroups based on urinary GA concentrations: low excretors (GA in urine < 100 mmol/mol Cr) and high excretors (GA in urine > 100 mmol/mol Cr) (Busquets et al., 2000). Both subtypes show similar clinical progression with an increased risk for striatal injury if untreated (Christensen et al., 2004; Kolker et al., 2006). There are three phenotypic presentations for GA1, asymptomatic, motor disability, and insidious onset (Strauss et al., 2007). In general, neonates present as asymptomatic, but some present with macrocephaly and frontotemporal atrophy. Affected individuals may present with brain hypoplasia and basal ganglia lesions, which in most cases, are triggered by an acute infection, fever and/or vomiting (Strauss et al., 2003; Bijarnia et al., 2008). Chronic progressive cerebral deterioration and acute encephalopathic crises accompanied by seizures, coma, and basal ganglia degeneration occur in two-thirds of untreated patients and are always followed by motor and neurological manifestations, including dyskinesia, dystonia, and hypotonia (Guerreiro et al., 2020). GA1 therapy consists of a low lysine, tryptophan diet, L-carnitine supplementation, and prompt management of intercurrent illnesses (Kolker et al., 2007). If not promptly and properly treated, severe morbidity and mortality can be caused by the irreversible neurological damage (Lindner et al., 2006). Asymptomatic neonates can be identified through expanded newborn screening (NBS) using tandem mass spectrometry (MS/MS), which has become a disease-changing intervention for GA1 (Boy et al., 2018).

In this study, we evaluated the clinical, biochemical, neuroradiological, and genetic features and outcomes of 101 Chinese patients with GA1 in an effort to expand our understanding of the variant spectrum and corresponding clinical manifestations of these patients.

## Materials and Methods

### Patients

A total of 101 patients (60 males and 41 females) with GA1 who were followed up in the Shanghai Xinhua Hospital between December 2007 and July 2019 were enrolled in this study. Patients were between 9 days and 11.2 years of age, and the demographic, clinical, and laboratory data were collected from patient charts. Written informed consent was obtained from the parents of study participants and the study protocol was approved by the Ethics Committee of the Xinhua Hospital Affiliated with Shanghai Jiao Tong University School of Medicine (No. XHEC-D-2020-153).

### Biochemical Analysis

Tandem mass spectrometry was used to evaluate amino acid and acylcarnitine concentrations in dried blood spots using a tandem mass spectrometer (API 4000, American Bio-Systems Inc.) (Han et al., 2015). Gas chromatography and mass spectrometry (GC-MS) was conducted on a GCMS-QP 2010 (Shimadzu Corporation, Kyoto, Japan) and was used to detect the concentration of organic acids, including glutaric acid in the urine, using a previously established protocol (Luo et al., 2003).

### Genetic Analysis

Genomic DNA was isolated from the peripheral blood of patients and their parents using a TIANamp Blood DNA Kit (Tiangen Biotech Co. Ltd., Beijing, China), following the manufacturer’s instructions. All 12 coding exons and flanking regions of the *GCDH* gene were amplified using polymerase chain reaction (PCR) as previously described (E et al., 2017), and these PCR products were sequenced on an ABI3700 sequencer (Applied Biosystems, Foster City, California, United States) after purification. The pathogenicity of novel variants was evaluated using the American College of Medical Genetics and Genomics (ACMG) standards and guidelines (Richards et al., 2015). The pathogenicity of the missense variants was predicted by Mutation Taster^1^, PolyPhen-2^2^, and SIFT^3^.

### Disease Diagnosis

Glutaric aciduria type 1 diagnosis was made based on the presence of C5DC in the blood and the C5DC/capryloylcarnitine (C8) ratio detected by MS/MS. In addition, urine GA levels detected by GC–MS as previously reported (E et al., 2017), together with the clinical features and conventional laboratory tests were used for diagnostic purposes. 88 patients’ diagnoses were confirmed by *GCDH* gene analysis. Patients diagnosed from the NBS program could be asymptomatic.

### Treatment

Metabolic maintenance treatment designed in accordance with the guideline recommendations (Boy et al., 2017b) consisted of the following therapies: (1) A lysine-free, tryptophan-reduced amino acid supplement for all patients up to 6 years of age; (2) After 6 years, a low lysine content natural protein diet avoiding lysine-rich foods was recommended; (3) Lifelong oral carnitine supplementation. Emergency treatment requires specialized nutritional products and medication.

### Outcome Evaluations

Clinical follow-up parameters were used to assess basic motor function and language development. Based on prognosis, the patients were divided into two main groups–normal and abnormal. The normal group showed no significant disability in daily life while the abnormal group presented with either deficits and/or delayed achievement of motor and/or speech milestones, including the need for support when walking and being unable to communicate or pronounce words effectively.

### Statistical Analysis

Statistical analysis was performed using Prism software (GraphPad, version 7.0). Continuous variables were not normally distributed; therefore, they were reported as the median and interquartile range. *Mann–Whitney U* and *Kruskal–Wallis H* tests were used to compare continuous variables between groups if the variables were non-parametric. *Chi-squared tests* were used to compare the categorical variables between groups.

## Results

### Clinical Features

This study recruited 101 individuals with a confirmed GA1 diagnosis from 22 provinces across China. The top three provinces were Jiangsu, Anhui, and Shandong, accounting for 43.6% of all the GA1 patients, followed by Fujian province accounting for 13.13% of all the GA1 patients. A total of 20 (19.8%) affected individuals (12 males and 8 females) were identified using NBS screening. Of the remaining 81 (80.2%) clinical patients, 78 (96.3%) were symptomatic with a median age at onset of 6.6 months (range 9 days–53.7 months) and a median age at diagnosis of 14.5 months (range 1.2–99 months). A total of 32 of the clinical patients (39.5%) had their first crises before the age of 24 months.

The clinical characteristics of all 101 patients are summarized in Table 1. Macrocephaly was the most common symptom, with this being the sole symptom in five patients, followed by movement disorders and seizures. Phenotypically, 20 patients (19.8%) were asymptomatic, 46 patients (45.5%) had at least one acute encephalopathic crisis presenting as seizures or vomiting, while 35 individuals (34.7%) presented as insidious onset and developed neurological disease and striatal injury in the absence of encephalopathic crises. One of these patients was a 3-year-old with recurrent headaches.

**TABLE 1:** Clinical features and MRI findings of patients with Glutaric aciduria type 1 (GA1).

Clinical features (*N* = 101)	*n*	%	MRI findings (*N* = 59)	*n*	%
Macrocephaly	45	44.6%	Wide sylvian fissures	28	47.5%
Movement disorders	42	41.6%	Abnormal signal of bilateral basal ganglia	21	35.6%
Seizure	40	39.6%	Leukoencephalopathy	17	28.8%
Mental retardation	35	34.7%	Wide ventricle	16	27.1%
Muscular hypotonia	32	31.7%	Cerebral atrophy	14	23.7%
Coma	11	10.9%	Arachnoid cysts	12	20.3%
Feeding difficulty	22	21.8%	Hydrocephalus/subdural effusion	8	13.6%
Vomiting	24	23.8%	Subdural hematomas	4	6.8%
Failure to thrive	14	13.9%	Myelination delay	2	3.4%
Diarrhea	14	13.9%	Abnormal brainstem/cerebellum signaling	2	3.4%
Jaundice	12	11.9%	Normal	1	1.7%
Abnormal respiration	7	6.9%			

### MRI Findings

A total of 59 patients were evaluated by MRI brain scans (Table1) with a median test age of 1 year (range 1 month–7.43 years), 55 of whom (93.2%) were clinical patients and four (6.8%) were asymptomatic (diagnosed by NBS). A total of 58 patients presented with abnormal images. The results demonstrated that widened sylvian fissures of the frontotemporal lobes was the most common symptom of this disease, followed by abnormal signals from the basal ganglia and leukoencephalopathy. Eight patients received a second MRI scan within 1 year, and while seven showed no changes or progress, one patient presented with a slightly smaller arachnoid cyst compared to that in their previous scan.

### Biochemical Results

The serum C5DC, C5DC/C8 ratio, and urinary GA values for each group of patients before and after medical intervention are summarized in Table 2. All 101 patients received at least one MS/MS test, and 89 patients were available for GC-MS analysis, with 78 patients identified as high excretors. There were significant differences in the C5DC concentration, C5DC/C8 ratio, and urinary GA levels in each of the patient groups following treatment. In addition, when these variables were compared between the NBS and clinical groups, significant differences were noted in all of them with the exception of the C5DC value in NBS patients. On comparing the NBS and clinical groups, only a significant difference in the C5DC values after treatment could be identified. The C5DC levels of three patients were normal at their initial evaluations and their GA1 diagnosis were based on an increased C5DC/C8 ratio, which was then further confirmed by a *GCDH* gene test.

**TABLE 2:** Levels of C5DC in the blood, C5DC/C8 ratios and urinary GA in each of the three groups.

	C5DC (μmol/L)		C5DC/C8		GA (mmol/mol Cr)	
			
**Reference range**	0.02∼0.25		0.10∼2.50		0∼8	
	Before treatment	After treatment	Before treatment	After treatment	Before treatment	After treatment
**All patients**	0.92 (0.11∼5.13)	0.86 (0.06∼4.68)	22.04 (1.6∼218)	11.86 (0.46∼73.45)	562.76 (0.12∼4,514)	201 (0.96∼1,099)
**NBS patients**	1.64 (0.11∼3.2)	1.77 (0.75∼4.68)	19.81 (1.6∼214)	12.54 (2.71∼40.57)	547.51 (1.92∼2,477)	230.85 (45.59∼378.82)
**Clinical patients**	0.83 (0.13∼5.13)	0.60 (0.06∼4.49)	22.29 (2.58∼218)	11.712 (0.46∼73.45)	578 (0.12∼4,514)	157.7 (0.96∼1,099)
*P*_(NBS–Clinical)_	0.148	0.002	0.973	0.657	0.947	0.702
*P*_(all patients)*_	0.012		<0.001		<0.001	
*P*_(NBS)*_	0.477		0.011		0.012	
*P*_(Clinical)*_	0.014		<0.001		<0.001	

*^∗^*P* value for each group before and after treatment.*

*NBS, newborn screening.*

The details of the nine patients with unmatched biochemical and clinical manifestations are listed in Table 3. Patient Nos. 1 and 2 were siblings and patient Nos. 3 and 4 were siblings, while the rest of the patients were unrelated. All the relevant biochemical indices were significantly elevated in patient Nos. 1–4, but their clinical characterizations were reasonably mild. In contrast, patient Nos. 5–9 showed only moderate increase in their biochemical indices but had much more severe clinical presentations than patient Nos. 1–4.

**TABLE 3:** Phenotype and biochemical results for nine patients with unmatched manifestations.

No.	Age (year)	Clinical manifestation	Before treatment			After treatment			Variation 1	Variation 2
				
			C5DC	C5DC/C8	GA	C5DC	C5DC/C8	GA		
**1**	0.6	Unable to raise her head and turn over at 3 months old following a severe fever	1.49	23.23	1,000	1.09	23.43	612.94	c.646-4_639 del CCAGGATC	c.1244-2 A>C
**2**	5.2	Normal	2.44	65	1,313.15	3.36	29.66	662.71	c.646-4_639 del CCAGGATC	c.1244-2 A>C
**3**	1.8	Unable to walk or speak after 18 months of age	2.36	12.54	284.20	1.16	47.57	157.7	c.452C>T (p.P151L)	c.873delC (p.N291Kfs*41)
**4**	8.2	Had drainage of hydrocephalus at the age of 8 months	0.31	25.89	368.20	1.57	18.32	426.7	c.452C>T (p.P151L)	c.873delC (p.N291Kfs*41)
**5**	0.7	Had a seizure at the age of 8 months	0.41	7.64	16.91	0.10	1.46	2.6	c.1169G>A (p.G390E)	c.406G>T (p.G136C)
**6**	0.7	Hypotonia in both legs at the age of 8 months	1.04	6.43	129.26	0.99	11.93	16.84	c.109_110delCA (p.Q37Efs*5)	c.554G>A (p.G185E)
**7**	0.5	Had seizures and vomiting at 6 months old; unable to raise her head at the age of 19 months	0.26	3.04	40.20	0.20	1.76	2.02	c.755G>A (p.G252D)	c.533G>A (p.G178E)
**8**	0.5	Hypotonia and unable to raise her head at 6 months old	0.45	9.06	14.09	0.24	3.2	2.53	c.755G>A (p.G252D)	c.1244-2 A>C
**9**	1.2	Had seizures and regressed movement at 14 months of age	0.52	–	7.88	0.183	14.98	5.92	c.908G>A (p.G303A)	c.1045G>A (p.A349T)

### Genetic Analysis and Genotype-Phenotype Correlations in GA1

**FIGURE 1:**
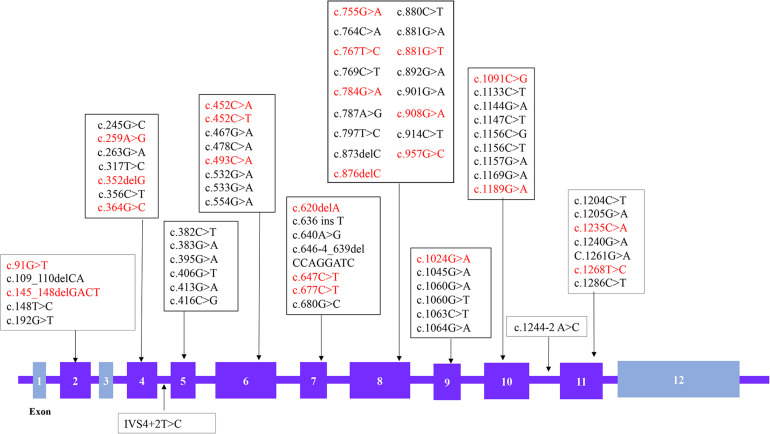
Distribution of the *GCDH* variations identified in 88 Glutaric aciduria type 1 (GA1) patients. The boxes represent the exons and novel variations are shown in red (The chromosome position is chr19:12,891,160-12,899,999 and the number of transcription version is NM-000159.3).

A total of 88 patient samples (87.1%) were sequenced for *GCDH*. As a result, we found that 87 patients were compound heterozygotes or homozygotes, while only one patient had a single variant. A total of 74 different variants were identified (Figure 1), including 61 missense variants (82.4%), six frameshifts (8.1%), five nonsense (6.8%), and two splicing (2.7%) mutations. The most frequent variant was c.1244-2A > C (18.3%), followed by c.1064G > A (p. Arg355His) (7.4%), c.533G > A (p. Gly178Glu) (3.5%), and c.1147C > T (p. Arg383Cys) (2.5%). A total of 17 patients carried homozygous variants, and nine of these carried the c.1244-2A > C variant. In addition, 23 novel variants were identified, including 18 missense variants, four frameshifts, and one nonsense variant. Among these, 17 of the novel missense variants were predicted to be pathogenic using Mutation Taster SIFT and polyphen2. Variant c.493C > A (p. Leu165Met) was predicted to be a neutral mutation when evaluated by SIFT, but was shown to be pathogenic using the other two algorithms. The genotype-phenotype correlation analysis focused on the four most common variants: c.1244-2A > C, c.1064G > A (p. Arg355His), c.533G > A (p. Gly178Glu), and c.1147C > T (p. Arg383Cys), and 47 patients carrying these variants were analyzed (Table 4). *Kruskal–Wallis test* showed that all four variants exhibited no significant difference for age of onset/diagnosis, clinical manifestations, biochemical results, or outcomes. An acute encephalopathic crisis was identified in one patient with c.1147C > T (p. Arg383Cys) (20%), one with c.533G > A (p. Gly178Glu) (14.3%), one with c.1064G > A (p. Arg355His) (25%), and four with c.1244-2A > C (17.4%). All the patients were classified as high excretors, except one, who carried a c.1244-2A > C variation. Taken together, these results suggest that there were no significant differences in biochemical or clinical phenotypes for these four genotypes.

**TABLE 4:** Correlation between genotype and phenotype in GA1 patients: comparison of the four most common variants from 47 patients.

	c.533G > A		c.1064G > A		c.1147C > T		c.1244-2A > C		*P* value
	n	%	n	%	n	%	n	%	
Case	7	14.9	12	25.5	5	10.6	23	48.9	
Age of onset (months)	5.6 (5∼6)		5.9 (0∼17.3)		9.25 (4∼14.5)		7.3 (0.3∼24.3)		0.735
Age of diagnosis (months)	7.9 (2∼66)		14.95 (0.7∼89.8)		5.8 (3.4∼42.8)		16.5 (0.9∼110.8)		0.454
NBS	2	28.6	1	8.3	2	40.0	5	21.7	0.484
**Nervous system**									
Macrocephaly	2	28.6	3	25.0	1	20.0	9	39.1	0.758
Movement disorder	3	40.0	7	60.0	2	40.0	9	40.0	0.742
Muscular hypotonia	3	40.0	7	60.0	1	20.0	8	30.0	0.426
Mental retardation	3	40.0	7	60.0	1	20.0	8	30.0	0.426
Seizure	4	60.0	5	40.0	1	20.0	11	50.0	0.613
**Gastrointestinal system**									
Diarrhea	1	20.0	0	0.0	1	10.0	2	10.0	0.682
Failure to thrive	0	0.0	0	0.0	1	10.0	4	20.0	0.458
Vomiting	1	20.0	1	10.0	2	20.0	2	10.0	0.860
Feeding difficulty	0	0.0	2	30.0	4	30.0	5	20.0	0.521
**Biochemical results (before treatment)**									
C5DC (μ mol/L)	1.04 (0.26∼3.37)		2.08 (0.38∼5.13)		1.49 (0.94∼2.33)		1.00 (0.11∼4.48)		0.826
C5DC/C8	15.64 (3.04∼19.43)		30.18 (4.45∼51.54)		22 (12.42∼45.91)		28.63 (2.7∼161.4)		0.251
GA (mmol/molCr)	404.69 (40.2∼1146.3)		409.17 (8.79∼1550.19)		742 (511.53∼1093.04)		773.44 (14.09∼2857.3)		0.402
Outcome									0.491
Normal	2		2		3		8		
Abnormal	4		8		2		13		
NA	1		2				2		

*NBS, newborn screening; NA, not available.*

### Outcomes

**FIGURE 2:**
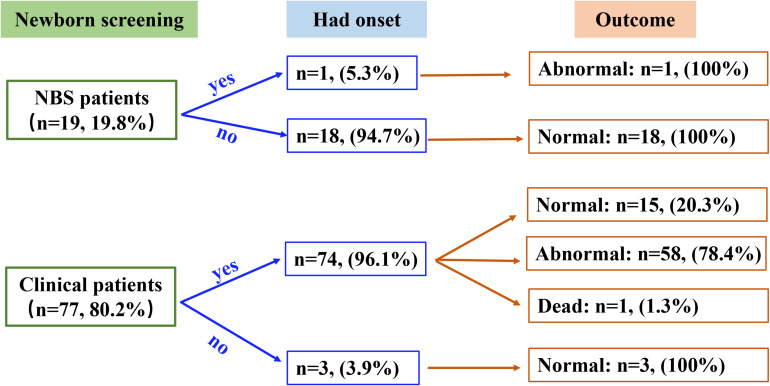
Summary of the newborn screening status, disease onset, and clinical outcome data for the participants in this study.

All patients received appropriate therapy from the time of diagnosis. The follow-up study was performed on 96 patients (Figure 2), as the other five patients discontinued the trial. Of the 19 NBS patients, 18 were asymptomatic at diagnosis and continued to develop normally, only one patient showed a slight delay in motor milestones without any acute encephalopathic crises and was unable to walk at 3 years of age. A total of 77 patients were in the clinical group, while three asymptomatic patients were diagnosed due to the presence of a GA1 sibling. Outcomes in the symptomatic patients varied, 58 presented with motor and/or speech dysfunctions, one patient died at 18 months of age and the remaining 15 developed normally. This data shows that NBS and clinical patient outcomes are significantly different (*P* < 0.001).

Of the 78 high excretor patients, 30 (38.5%) developed normally, 43 (55.1%) became disabled, and one died (four patients were lost to follow-up). Similar results were found in the 11 low excretor patients–four (36.4%) developed normally, while seven (63.6%) had movement and/or speech impediments (*P* > 0.05).

## Discussion

Our study was designed to systematically investigate the clinical, biochemical, and genetic characteristics of 101 GA1 patients from mainland China ultimately identifying 23 novel variants. Unlike Turkish and Indian patients with GA1 (Radha Rama Devi et al., 2016; Gurbuz et al., 2020), none of our patients were from consanguineous families. Approximately 50% of the study participants were from Jiangsu, Shandong, Anhui, and Fujian Province, with Jiangsu being the most common. Studies have shown that GA1 prevalence is higher in the Fujian province (Lin et al., 2019). However, our data, derived from Shanghai, might be influenced by several geographical factors, including the fact that Jiangsu is much closer to Shanghai making it more convenient for these patients.

Typically, GA1 presents in infancy after an acute metabolic encephalopathy that results in striatal necrosis, such as dystonia, mostly between the ages of 3 and 36 months, while some children only present with macrocephaly during their neonatal development (Strauss et al., 2003; Boy et al., 2017b). The onset of dystonia may be caused by catabolism and infectious diseases (Kolker et al., 2006; Harting et al., 2009). Our study population was made up of patients with acute onset (45.5%) and insidious onset (34.7%). The most common presentation for our acute onset patients were seizures, vomiting, and/or diarrhea. This is in agreement with previous studies, which suggest that seizures could be the initial clinical presentation of GA1 and that these patients experience a higher frequency of epilepsy (Young-Lin et al., 2013; Kolker et al., 2015). This was confirmed by our data that demonstrated that 40 patients experienced single or recurrent seizures and that in 28 of these patients, seizures were the first symptom. Macrocephaly is the most common characteristic of GA1 and was found in nearly half of the patients in this cohort; however, this value is lower than that reported in other studies (Bjugstad et al., 2000; Renaud, 2012). In addition, movement disorders and mental retardation were common in this cohort. Although the severity of the acquired movement disorders and motor disabilities remained stable with increasing age, dystonia tended to become fixed and was associated with the development of parkinsonism with increasing age (Tuncel et al., 2018). In addition to acute and insidious onset patients, there have been several reports of patients with late onset symptoms (Pierson et al., 2015; Boy et al., 2017a). This late-onset type has been used to describe patients diagnosed after 6 years of age, who present with a variety of non-specific neurological symptoms, including headaches, nausea, and muscular weakness (Tuncel et al., 2018; Gelener et al., 2020). Our data set included three late onset patients (aged from 6.4 to 11.2 years), all of whom were diagnosed because of their GA1 siblings and showed normal development. Two of these patients underwent an MRI scan and both resented with white matter changes. Since neurological signs are also common in late onset GA1 patients, brain MRI evaluations can help with diagnosis.

Half of the macrocephaly patients received MRI evaluations with the majority of the reports finding striatal changes, subdural collection, and widening of both sylvian fissures (Mohammad et al., 2015). We found that sylvian fissure widening, also known as Batwing sign, was the most common presentation. However, a large proportion of our patients underwent brain MRIs under 1 year of age. During this period, the widening of the cerebral operculum is related to underdevelopment rather than atrophy or the absence of the cerebral gyri comprising the operculum (Twomey et al., 2003; Strauss et al., 2007). Therefore, re-examining the brain MRIs of these patients after 1–2 years should be required. The second most common MRI finding was abnormal signaling in the bilateral basal ganglia, and injuries within this brain region are the main determinant of morbidity. More than 50% of the patients in this study who presented with bilateral basal ganglia abnormalities experienced seizures, and nearly 40% of these patients developed movement disorders. The third most common MRI characteristic was leukoencephalopathy, which is not a specific manifestation in GA1; however, it is an important symptom in most cerebral organic acid disorders (Sauer et al., 2011; Kölker et al., 2013). The neuropathological implications of the presence of white matter abnormalities seem to focus on spongiform myelinopathy, which is probably caused by the toxin accumulation associated with GA1 resulting in desmyelination or demyelination (Gerstner et al., 2005; Zinnanti et al., 2006). In addition, subdural hematomas, which might be caused by stretched cortical veins and venous hypertension (Hou et al., 2007; Strauss et al., 2010), were identified in three patients. Notably, the frequency of arachnoid cysts was higher in our data, and when this non-specific neurological sign is found in conjunction with frontoparietal brain atrophy and widened sylvian fissures, it may reflect abnormal brain growth during intrauterine development. There are some limitations to these results because most of the MRI results came from different hospitals across the country suggesting that there may be some differences in the interpretation of the scans.

Glutaric acid 1 is a treatable neurometabolic disorder when diagnosed in early neonates (Pusti et al., 2014). Our study showed that the outcomes were much better in patients diagnosed by NBS, who were able to receive intensive management interventions. In contrast, clinical patients showed a higher frequency of complications and reduced life expectancy. It has been demonstrated that the onset of encephalopathic crises is the predictable outcome of GCDH deficiency (Strauss et al., 2003; Kyllerman et al., 2004). Although only one patient died in this cohort, the disability rates were frustrating with more than 80% of the clinical patients presenting with motor and/or speech disabilities. While in NBS patients, these disability rates were significantly decreased. Thus our study confirmed the findings of several others and demonstrate that NBS is a beneficial, disease-changing intervention for GA1 and that early intervention is critical for preventing irreversible central nervous system (CNS) damage associated with GA1 (Viau et al., 2012; Lee et al., 2013; Boy et al., 2018). To our knowledge, GA1 has been included in the NBS plans in many of the provinces across mainland China, Taiwan province, and Hongkong, but increased vigilance is needed, since some low excretors can be missed by NBS. Therefore, further testing and gene analysis is necessary.

A definite diagnosis of GA1 relies on biochemical and genetic analyses. In this study, nearly 90% of the patients were high excretors and their clinical outcomes were similar to the low excretors, which was consist with previous studies (Christensen et al., 2004). Of note, GA1 diagnosis was usually based on C5DC, GA, and 3-OH-GA (Boy et al., 2017b; Shaik et al., 2019) concentrations; however, our previous study demonstrated that the C5DC/C8 ratio is also important in the diagnosis of GA1 and might be more sensitive than C5DC (E et al., 2017). As GC–MS is not included in NBS, some NBS patients were diagnosed on the basis of their MS/MS results and re-examined using an additional MS/MS and GC–MS analyses. Thus, when the first round of MS/MS results demonstrate abnormal or slightly increased C5DC, a positive diagnosis of GA1 should be considered especially where the C5DC/C8 ratio suggests it as a possibility. In addition, all patients should undergo a *GCDH* gene analysis.

A total of 88 patients underwent *GCDH* gene sequencing, and we identified 74 unique variants, most of which were classified as missense variants. Pathogenic variants responsible for GA1 vary between ethnic groups. The c.1204C > T (p.Arg402Trp) is the most common in the European population (Christensen et al., 2004), and it has been reported that c.1244-2A > C was a recurrent variant in the Chinese population both in mainland China and Taiwan province (Tang et al., 2000). This was further confirmed by our study where the c.1244-2A > C accounted for 18.3% of variant alleles and resulted in a splicing error. The variants identified in this study were widely distributed throughout the gene, with the largest number in exon 8. Besides c.493C > A (p.Leu165Met), all 17 other novel missense variants were predicted to have some pathogenic potential when evaluated by Mutation Taster, SIFT, and Polyphen2. p.Leu165Met was a compound heterozygous variant found in a patient who carried another novel variant, c.91G > T (p.Glu31Ter). This patient was a high excretor who experienced their first crisis before 12 months of age and who went on to develop mental retardation. It has been postulated that p.Arg402Trp and p.Ala293Thr were most frequently identified in high excretors, while p.Val400Met and p.Arg227Pro are only found in low excretors (Busquets et al., 2000; Mühlhausen et al., 2003; Funk et al., 2005; Gallagher et al., 2005). Although p.Arg402Trp accounts for 40% of alleles in patients of German origin (Radha Rama Devi et al., 2016), it was less common in our study. p.Ala293Thr and p.Val400Met were not detected in our study, but we did find p.Arg402Trp and p.Arg227Pro in both our high excretor and low excretor patients. However, we could not determine a clear correlation between these variants and biochemical phenotype. In addition, we identified several unmatched phenotype-biochemical relationships that should be evaluated in the future: c.646-4_639 del CCAGGATC and c.452C > T, which may be related to high excretors who present with a milder phenotype, while c.109_110delCA, c.755G > A, and c.908G > A may be related to low excretors who present with more severe phenotypes. To explore these genotype-phenotype relationships, we analyzed the four most common variants, with each identified in at least three cases. The distribution of the clinical and biochemical phenotypes was similar in all groups and no differences in outcome were noted amongst these variants. Researchers have tried for years to find a link between the genotype and phenotype of these patients in an effort to predict their prognosis with limited success (Sanju et al., 2021; Sitta et al., 2021). Recently, an Egyptian study suggested that six variants: p.Trp50Arg, p.Glu64Asp, p.Ser119Leu, p.Arg128Gln, p.Ser139Leu, and p.Arg402Trp, might present with a genotype-phenotype correlation, indicating a poor outcome (Mosaeilhy et al., 2017). Notably, five of these variants were detected in our study, and most patients carrying these variants did become disabled. However, we cannot draw a similar conclusion as our sample size remains relatively small. More patients should be recruited so that we can explore the relationships between phenotype and genotype.

In conclusion, a cohort of 101 Chinese patients with GA1 were analyzed and genotyped. Our findings demonstrated that macrocephaly was the most common presentation of GA1, and C5DC/C8 ratios should be used in the diagnosis of GA1. We could not draw any clear genotype-phenotype correlations due to the high number of variants and the relatively limited number of patients. However, our study has expanded the spectrum of genotypes for Chinese patients with GA1. Our data supports the expansion of the NBS and genotyping programs for GA1 in an effort to promote early diagnosis and improved clinical outcomes.

## Data Availability Statement

The original contributions presented in the study are included in the article, further inquiries can be directed to the corresponding author.

## Ethics Statement

The studies involving human participants were reviewed and approved by Ethics Committee of the Xinhua Hospital Affiliated with Shanghai Jiao Tong University School of Medicine (No. XHEC-D-2020-153). Written informed consent to participate in this study was provided by the participants’ legal guardian/next of kin. Written informed consent was obtained from the minor(s)’ legal guardian/next of kin for the publication of any potentially identifiable images or data included in this article.

## Author Contributions

LH initiated and designed the study. XG contributed to the conception of the study. HZ, WQ, and JY contributed to the acquisition and analysis of data. FX and ZG contributed to the testing of MS/MS and GC/MS. HE and LL contributed to drafting the text, preparing the figures and tables, they contributed equally to the work. All authors contributed to the article and approved the submitted version.

## Conflict of Interest

The authors declare that the research was conducted in the absence of any commercial or financial relationships that could be construed as a potential conflict of interest.

## Footnotes

1 http://www.mutationtaster.org/

2 http://genetics.bwh.harvard.edu/pph2

3 http://sift.jcvi.org/www/SIFT_enst_submit.html
